# 

**Published:** 1898-08

**Authors:** 


					﻿Vol. xviii.
AUGUST, 1898.
No^7
THE lSUR
HOMEOPATHIC pHY^lCPAfJ,
A Monthly Journal of Homoeopathic Materia Medica
and Clinical Medicine.
“ He i» a freeman whom truth makes free, and all are slaves beside."
"Seek the Truth: come whence it may, cost what it wilL."
BDITED AND PUBLISHED BY
WALTER M. JAMES, M. D.
PHILADELPHIA:
JSTo. 1231 LOCUST STREET.
^Yearly S^bicrtptwn, $2.50, invariably in advan.ee, Single nuvnberv9 25
Entered at the Philadelphia Peet-Office aa Beoond-Claai Mail Matter.
				

